# Culturable Bacterial Community on Leaves of Assam Tea (*Camellia sinensis* var. *assamica*) in Thailand and Human Probiotic Potential of Isolated *Bacillus* spp.

**DOI:** 10.3390/microorganisms8101585

**Published:** 2020-10-14

**Authors:** Patthanasak Rungsirivanich, Witsanu Supandee, Wirapong Futui, Vipanee Chumsai-Na-Ayudhya, Chaowarin Yodsombat, Narumol Thongwai

**Affiliations:** 1 Department of Biology, Faculty of Science, Chiang Mai University, Chiang Mai 50200, Thailand; patthanasak_bas@hotmail.com (P.R.); wirapong49187@gmail.com (W.F.); vipaneec@gmail.com (V.C.-N.-A.); 2 Graduate School, Chiang Mai University, Chiang Mai 50200, Thailand; 3 Darunsikkhalai School, King Mongkut’s University of Technology Thonburi, Bangkok 10150, Thailand; witsanu.sup@mail.kmutt.ac.th; 4 Production and Water Quality Control Division, Provincial Waterworks Authority Region 2, Saraburi 18000, Thailand; chaowarin6637@gmail.com; 5 Research Center in Bioresources for Agriculture, Industry and Medicine, Chiang Mai University, Chiang Mai 50200, Thailand

**Keywords:** antibiotic resistance, bacterial adhesion, diversity, Miang, microecosystem, probiotics, surface hydrophobicity, Vero cells

## Abstract

Assam tea plants (*Camellia sinensis* var. *assamica*) or Miang are found in plantations and forests of Northern Thailand. Leaf fermentation has been performed for centuries, but little information is available about their associated microbial community. One hundred and fifty-seven bacterial isolates were isolated from 62 Assam tea leaf samples collected from 6 provinces of Northern Thailand and classified within the phyla of Firmicutes, Actinobacteria, and Proteobacteria. Phayao and Phrae provinces exhibited the highest and the lowest bacterial diversities, respectively. The bacterial community structural pattern demonstrated significant differences between the west and the east sides. Since some *Bacillus* spp. have been reported to be involved in fermented Miang, *Bacillus* spp. isolated in this study were chosen for further elucidation. *Bacillus siamensis* ML122-2 exhibited a growth inhibitory effect against *Staphylococcus aureus* ATCC 25923 and MRSA DMST 20625, and the highest survival ability in simulated gastric and intestinal fluids (32.3 and 99.7%, respectively), autoaggregation (93.2%), cell surface hydrophobicity (50.0%), and bacterial adherence with Vero cells (75.8% of the control *Lactiplantibacillus* *plantarum* FM03-1). This *B. siamensis* ML122-2 is a promising probiotic to be used in the food industry and seems to have potential antibacterial properties relevant for the treatment of antibiotic-resistant infections.

## 1. Introduction

Assam tea (*Camellia sinensis* var. *assamica*) or “Miang”, as called by Northern Thai people, is a perennial native plant from the highlands of Northern Thailand, i.e., Chiang Mai, Chiang Rai, Lampang, Nan, Phayao, and Phrae provinces, and neighbor countries [1,2]. “Fermented Miang” production has been inherited from generation to generation for centuries [3]. The microbiome of fermented Assam tea leaves studied by several researchers has revealed the involvement of naturally-occurring lactic acid bacteria (LAB), including *Enterococcus calmelliae*, *Lacticaseibacillus casei*, *Lacticaseibacillus camelliae*, *Lacticaseibacillus pantheris*, *Lacticaseibacillus thailandensis*, *Lactiplantibacillus pentosus*, *Lactiplantibacillus plantarum*, *Leuconostoc mesenteroides*, *Lentilactobacillus buchneri*, *Levilactobacillus brevis*, *Pediococcus siamensis*, *Streptococcus faecalis*, and *Streptococcus lactis* [1,4,5]. Hence, fermented Assam tea leaves are rich in probiotics providing health benefits besides its catechin, catechin derivatives, and other bioactive compounds. 

Microbial diversity in tea plants and its influence on the fermentation process has been studied by several authors, indicating that the process is driven by the interaction between fungal and bacterial diversity, community composition, and environmental factors (climate, relative humidity, soil pH, and soil structure) [2,6,7,8,9,10].

The bacterial community on Assam tea leaves from the tea gardens in Nan province of Thailand revealed by Rungsirivanich et al. [11] consisted of the genera *Bacillus*, *Floricoccus*, *Kocuria*, *Lysinibacillus*, *Micrococcus*, and *Staphylococcus*. Additionally, *Bacillus licheniformis*, *Bacillus siamensis*, and *Bacillus tequilensis* were pointed as important elements for biological control during leaf fermentation by inhibiting the growth of food spoilage and pathogenic microbes [12]. In this gut microbiome era, sources of probiotics are intensely studied, and the Assam tea plant and its products have proved to be notable.

For decades, LAB have been widely recognized as probiotics, the live microbes conferring health benefits on the host when adequate amounts are administered [13]. Probiotic consumption presents several benefits such as prevention of diarrhea and bowel inflammation that leads to cancer. Besides that, probiotics also help to decrease cholesterol levels and improve lactose intolerance as well as to enhance the utilization of nutrients [14]. Recently, *Bacillus* spp. have been studied as spore-forming probiotics due to the presence of endospores that enhance tolerance to the extreme conditions of the gastrointestinal tract [15]. Previous studies also showed that some species of *Bacillus* participate in antimicrobial peptide production such as bacteriocin-like inhibitory substance (BLIS) [16,17].

This study aimed to assess the bacterial diversity on Assam tea leaves collected from six provinces in Northern Thailand, namely Chiang Mai, Chiang Rai, Lampang, Phayao, Phrae, and Nan provinces, and to investigate the probiotic properties of *Bacillus* spp. isolated from Assam tea leaf surfaces. The obtained results will provide insightful information regarding the role of these bacteria on Assam tea leaves as well as their probiotic potential.

## 2. Materials and Methods

### 2.1. Sampling Site and Collection of Assam Tea Leaves

**Figure 1:**
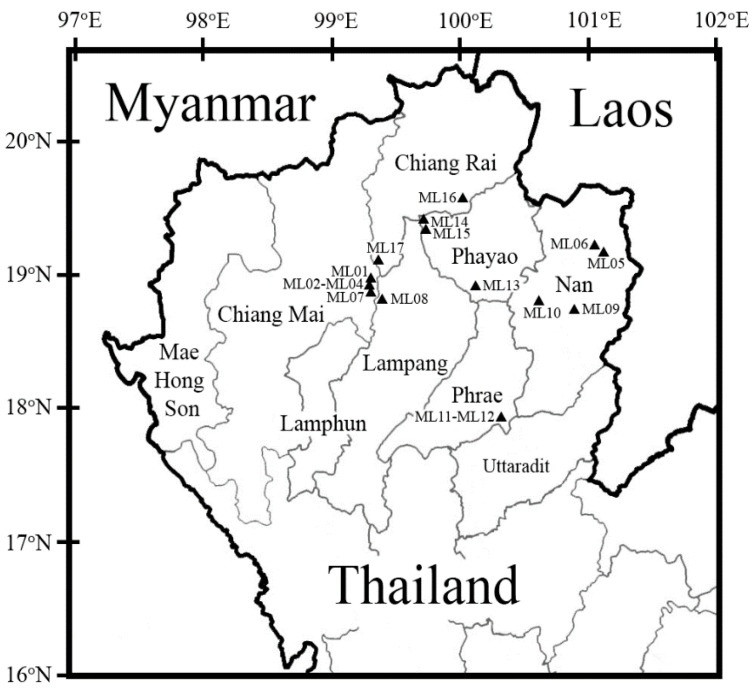
The areas indicating the Assam tea plant sampling sites in Northern Thailand including Chiang Mai, Chiang Rai, Lampang, Phayao, Phrae, and Nan provinces.

Sixty two fresh Assam tea leaf samples were collected during the time period of 7 July 2015 to 18 November 2018 at altitudes of 243–1278 m above sea level from 17 sampling sites (Figure 1 and Table S1) in Northern Thailand, including a province on the west side (Chiang Mai (codes ML01-ML04 in Doi Saket and ML07 in Mae On districts)) and five provinces on the east side (Chiang Rai (codes ML16 in Mae Loi and ML17 in Wiang Papao districts), Lampang (code ML08 in Mueang Pan district), Phayao (code ML13 in Dok Khamtai and ML14–ML15 in Mae Chai districts), Phrae (codes ML11–ML12 in Mueang Phrae district), and Nan (codes ML05-ML06 in Pua, ML09 in Phu Phiang, and ML10 in Mueang Nan districts)). The collection of Assam tea leaves was performed as described by Levetin and Dorsey [18] with slight modifications. A 4 cm^2^ (2 cm × 2 cm) area on the surface of fresh leaves was swabbed using a moistened sterile cotton swab. Bacterial isolation was performed as soon as possible within a day.

### 2.2. Isolation of Bacteria

A serial dilution (1 × 10^1^–1 × 10^2^) of each swabbed sample was conducted using NaCl, 0.85% (*w/v*). The spread plate technique was subsequently performed on tryptic soy agar (TSA) (Merck™, Darmstadt, Germany) and de Man, Rogosa, and Sharpe (MRS) agar (Merck™) containing 0.004% (*w/v*) bromocresol purple. The culture plates were incubated at 37 °C for 24–48 h. All bacterial colonies on TSA showing different morphologies and the yellow bacterial colonies on MRS agar were further re-streaked to obtain pure cultures [5,12]. These bacterial isolates were kept at −20 °C until used.

### 2.3. Bacterial Genomic DNA Extraction

The chromosomal DNA of each bacterial isolate was extracted according to the method described by Pitcher et al. [19] with slight modifications. Briefly, each isolate was grown in tryptic soy broth (TSB) at 37 °C for 24–48 h. One milliliter of culture broth was centrifuged for 5 min at 6000 rpm at 4 °C. The supernatant was removed, and the pellet was resuspended in 1 mL of 1X TNE buffer (10 mM Tris, 1 mM EDTA, 0.1 M NaCl, pH 7.4). The mixture was then centrifuged for 5 min at 6000 rpm at 4 °C. Three hundred microliters of 1X TNE containing 2% (*v/v*) Triton X-100 were added to each pellet and mixed for 2 min using a vortex mixer. Subsequently, 300 µL of a solution containing phenol:chloroform:isoamyl alcohol (25:24:1) was added and mixed for 2 min, followed by centrifugation for 10 min at 12,000 rpm at 4 °C. The aqueous phase was transferred to a new microtube and mixed with 500 µL of ethanol at 95% (*v/v*). Each tube was stored at –20 °C overnight and spun at 12,000 rpm at 4 °C for 15 min. The supernatant was slowly removed and the pellet was dried at room temperature and resuspended in 30–50 µL in sterile distilled water. The DNA on each tube was kept at −20 °C.

### 2.4. 16S rRNA Gene Amplification

Amplification of the bacterial 16S rRNA gene was performed by polymerase chain reaction (PCR) in a thermal cycler (Labcycler, SensoQuest, Germany). The universal bacterial primers, 27F (5′-AGA GTT TGA TCM TGG CTC AG-3′) and 1492R (5′-TAC GGY TAC CTT GTT ACG ACT T-3′) were used [20]. The PCR reactions were held at 94 °C for 5 min for DNA denaturation, followed by 35 cycles of denaturation at 94 °C for 30 s, annealing at 56 °C for 30 s, extension at 72 °C for 1 min, and an elongation step at 72 °C for 5 min to ensure complete amplification. PCR samples were temporarily stored at 4 °C. Amplicons were electrophoresed on agarose gel, 0.8% (*w/v*), supplemented with 1X nucleic acid staining solution (RedSafe^®^, iNtRON Biotechnology, Inc., Seongnam-Si, Korea) for 50 min at 95 V and 300 mA in 1X Tris-acetate-EDTA (TAE) buffer (40 mM Tris-acetate, 1 mM EDTA, pH 8.0) using the electrophoretic gel system (EC320, Minicell Primo, CA, USA) at room temperature. The marker used was a 1 kb DNA ladder (RBC Bioscience, New Taipei, Taiwan). The gel documentation system (SynGene, Frederick, MD, USA) was used for gel visualization under UV light. The PCR products were purified and sequenced by DNA sequencing services (First BASE Laboratories Sdn Bhd., Selangor, Malaysia). The 16S rRNA gene sequence homologies were retrieved from the National Center for Biotechnology Information (NCBI) GenBank (https://www.ncbi.nlm.nih.gov/) and EzBioCloud databases (https://www.ezbiocloud.net/). The sequence data were aligned, and the phylogenetic tree was constructed by a neighbor-joining method [21] with the MEGA 7 program [22]. The bootstrap values of 1000 replicates were performed [23] with the Tamura–Nei model [24]. Amplicon sequences were deposited in the GenBank.

### 2.5. Probiotic Property Characterization of Bacillus Strains

#### 2.5.1. Antibacterial Activity Investigation

The pathogenic bacteria consisting of *Bacillus cereus* TISTR 687, *Escherichia coli* ATCC 25922, *E. coli* O157:H7 DMST 12743, methicillin resistant *Staphylococcus aureus* DMST 20625, *Salmonella* Typhi DMST 22842, *Shigella dysenteriae* DMST 1511, *Staphylococcus aureus* ATCC 25923, and *Vibrio cholerae* DMST 2873 were used for determination of antibacterial activity. The *Bacillus* strains were cultured in TSB at 37 °C overnight. The supernatant was filtered through a 0.45 μm nylon syringe filter before use. Thirty-three microliters of the *Bacillus* culture filtrate were used to evaluate the antimicrobial properties against pathogenic bacteria using the agar well diffusion method [25].

#### 2.5.2. Antibiotic Susceptibility Assay

Antibiotic discs consisting of amikacin (30 μg), amoxyclav (30 μg), ampicillin (10 μg), ampicillin/sulbactum (10 μg/10 μg), cefixime (5 μg), ceftriaxone (30 μg), cefuroxime (30 μg), cefuroxime axetil (30 μg), cefotaxime (30 μg), cefoxitin (30 μg), co-trimoxazole (25 μg), gentamicin (10 μg), meropenem (10 μg), ofloxacin (5 μg), tetracycline (30 μg), and ticarcillin/clavulanic acid (75/10 μg) were purchased from Himedia, India. The susceptibility test of *Bacillus* strains was done as described by the Clinical Laboratory Standards Institute (CLSI) [26].

#### 2.5.3. Survival Evaluation in Simulated Gastric and Intestinal Fluids

The fluids resembling human gastric and intestinal fluids were prepared according to the method of Huang and Adams [27]. The *Bacillus* isolates were grown in TSB at 37 °C for 18–24 h before harvesting by centrifugation for 5 min at 6000 rpm at 4 °C. Then, the cell pellets were washed twice and resuspended in 1X PBS (~1 × 10^9^ CFU/mL). Then, the *Bacillus* cell suspension was inoculated into simulated gastric fluid (TSB supplemented with pepsin, 3 mg/mL, pH 2.5) to obtain about 1 × 10^8^ CFU/mL of the initial cell concentration. The mixture was incubated at 37 °C and 50 rpm in a shaker incubator. Viable cell counts were performed on TSA plates at 0, 1, 2, 3, and 4 h after incubation. In parallel, *Bacillus* isolates were inoculated into the simulated intestinal fluids (TSB added with pancreatin, 1 mg/mL, and bile salt, 0.3% (*w/v*), pH 8.0). Viable bacterial cell counts were done on TSA plates at 0, 1, 2, 4, 6, 12, and 24 h after incubation. The percentage of survival rate was calculated.

#### 2.5.4. Cellular Autoaggregation Assay

Autoaggregation was assessed following the modified protocol of Valeriano et al. [28]. To harvest the *Bacillus* grown cells, centrifugation at 6000 rpm 4 °C for 5 min was conducted. The cells were resuspended in 1X PBS to obtain the turbidity of 0.1 at 600 nm (OD_i_) and left standing undisturbed at room temperature. The absorbance of the upper suspension fluid was tested at 600 nm (OD_t_) at 1, 2, 4, 6, 12, 24, 36, and 48 h. The auto-aggregation percentage was estimated in accordance with the following equation: Autoaggregation (%) = (1 − (OD_t_/OD_i_)) × 100.

#### 2.5.5. Cell Surface Hydrophobicity Assay

Cellular hydrophobicity was elucidated by measuring the ability of bacterial cells to adhere to hydrocarbon [15]. The cells of *Bacillus* after cultivation at 37 °C for 18–24 h were harvested by centrifugation. The cell pellet was adjusted equivalent to 0.1 at 600 nm (A_initial_) (approximately 1 × 10^8^ CFU/mL) in 1X PBS. The resuspended cells and xylene (3 mL of each) were added into the tube and mixed well with a vortex mixer. The mixture was allowed to stand without disturbance for 30 min at room temperature to allow separation of organic and aqueous phases. Measurement of the aqueous phase final absorbance (A_final_) was conducted. The control used was *Lactobacillus acidophilus* TISTR 2365. The surface hydrophobicity percentage was calculated as the following: surface hydrophobicity (%) = ((A_intial_ − A_final_)/A_intial_) × 100.

#### 2.5.6. In Vitro Bacterial Adhesion Assay

Cell adhesion was carried out by using the Vero cell line, a kidney epithelial cell. The assay was performed according to the modified method of Llanco et al. [29]. The Vero cells were cultivated in 6-well plates with Dulbecco’s modified Eagle medium (DMEM, Gibco^®^, Loughborough, UK) containing 10% (*v/v*) fetal bovine serum (Capricorn Scientific GmbH, Ebsdorfergrund, Germany) and 1% (*v/v*) penicillin/streptomycin (Caisson Laboratories, Inc., Smithfield, UT, USA), and incubated at 37 °C in a CO_2_ incubator for 48 h. Afterwards, the harvested cells were adjusted equivalent to 1 × 10^5^ cell/well prior to incubation overnight at 37 °C in a CO_2_ incubator, and then triple washed with 1X PBS. The cell pellets of *Bacillus* strain were resuspended in DMEM without antibiotics before addition into the well (1 × 10^8^ CFU/well final concentration). The plate was incubated for 1 h and washed three times with 1X PBS. After that, the Vero cells were fixed in absolute methanol for 5 min prior to staining with Giemsa stain for 15 min. Control strains were *Escherichia coli* ATCC 25922 (known as normal gut flora) and the well-known probiotics *Lactobacillus acidophilus* TISTR 2365 and *Lactiplantibacillus plantarum* FM03-1 (GenBank accession no. MF599378). Bacterial adhesion characteristics were observed under a compound microscope, and the percentage of adhesion was elucidated

### 2.6. Statistical Analysis

Alpha diversity was conducted to analyze the complexity of family diversity on Assam tea leaves through four indices including Shannon’s index [30], Pielou’s evenness index [31], Simpson’s index [32], and species richness [33]. To examine the relationship between relative abundances of bacterial families and provinces, the number of an individual family found divided by the total number of families found in the province was multiplied by 100, and the histogram was constructed using Microsoft Excel software (version 1909) [7]. Principal coordinate analysis (PCoA) was performed using PAST software (version 3.25) [34]. A one-way analysis of variance (ANOVA) was performed to investigate statistical significance of antibacterial activity, survival in simulated gastrointestinal fluids, cellular autoaggregation, cell surface hydrophobicity, and bacterial adhesion assay using SPSS 22.0 with Duncan’s multiple range tests. *p* < 0.05 was considered to indicate significant difference.

## 3. Results

### 3.1. Bacterial Isolation from Assam Tea Leaves and 16S rRNA Gene Identification

**Figure 2:**
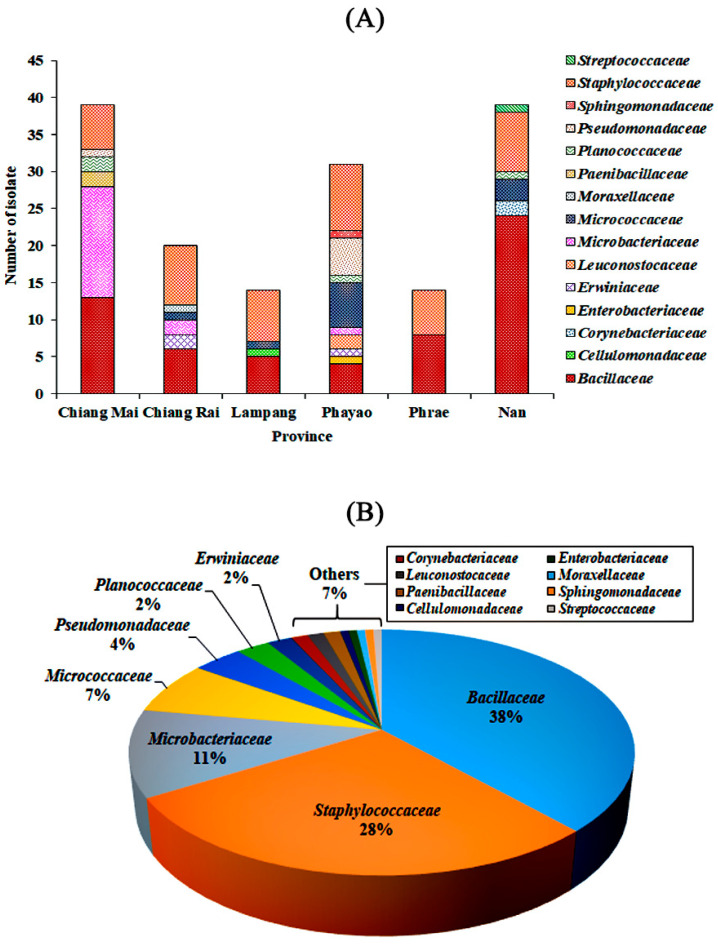
Bacterial families of all bacterial isolates obtained from Assam tea leaves collected from six provinces of Northern Thailand. (**A**) Number of bacterial isolates from each family in each province, (**B**) proportion of each bacterial family among the 157 isolates obtained.

Viable bacterial cells were obtained from 62 fresh Assam tea leaf surfaces collected in the 17 sampling sites (Figure 1; Table S1). The TSA grown colonies provided 156 bacterial isolates. A maximum of seven different colony morphologies were found in samples collected from Thep Sadej subdistrict, Doi Saket district, Chiang Mai province (code ML041; 18°55′17.81″ N, 99°19′53.91″ E). The number of bacterial isolates from each province were 39 = 39 > 30 > 20 > 14 = 14 obtained from Chiang Mai = Nan > Phayao > Chiang Rai > Lampang = Phrae provinces, respectively. Meanwhile, only one bacterial isolate with acid producing capacity but catalase negative was found on MRS agar from the sample collected in Sritoi subdistrict, Mae Chai district, Phayao province (code ML151; 19°21′13.31″ N, 99°42′48.01″ E;) (Figure 2A and Table S1).

All 157 bacterial isolates were found within three phyla (Actinobacteria, Firmicutes, and Proteobacteria), 15 families (*Bacillaceae*, *Cellulomonadaceae*, *Corynebacteriaceae*, *Enterobacteriaceae*, *Erwiniaceae*, *Leuconostocaceae*, *Microbacteriaceae*, *Micrococcaceae*, *Moraxellaceae*, *Paenibacillaceae*, *Planococcaceae*, *Pseudomonadaceae*, *Sphingomonadaceae*, *Staphylococcaceae*, and *Streptococcaceae*), and 20 genera (*Acinetobacter, Anoxybacillus, Bacillus, Brevibacillus, Cellulomonas, Corynebacterium, Curtobacterium, Enterobacter, Floricoccus, Kocuria, Lysinibacillus, Macrococcus, Microbacterium, Micrococcus, Pantoea, Pseudomonas, Solibacillus, Sphingomonas, Staphylococcus,* and *Weissella*) (Figure 2B and Figure S1).

The family *Bacillaceae* comprised the largest number of members (60 isolates). Interestingly, only two bacterial families, *Bacillaceae* and *Staphylococcaceae*, could be found in all provinces. Moreover, Phayao province displayed the highest number of bacterial families (10) including *Bacillaceae*, *Enterobacteriaceae*, *Erwiniaceae*, *Leuconostocaceae*, *Microbacteriaceae*, *Micrococcaceae*, *Planococcaceae*, *Pseudomonadaceae*, *Sphingomonadaceae*, and *Staphylococcaceae*, with 16S rRNA gene sequence similarity between 93.2 and 100.0%. For the MRS grown isolate, it was identified as a member of the family *Leuconostocaceae*, with 16S rRNA gene sequence similarity of 99.9% (Table S2).

### 3.2. Bacterial Community on Assam Tea Leaf Surface

**Figure 3:**
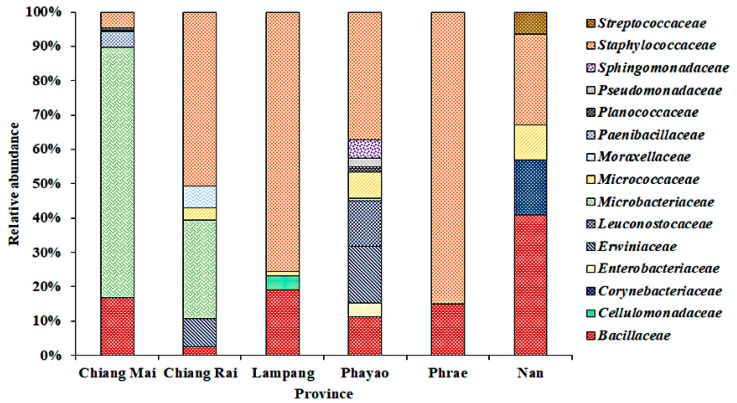
Relative abundance of different bacterial families found on Assam tea leaves collected from Chiang Mai, Chiang Rai, Lampang, Phayao, Phrae, and Nan provinces.

The relative abundance of bacterial families on Assam tea leaves from each province is presented in Figure 3. Leaves of Assam tea collected from Phayao province demonstrated the highest diversity with 10 bacterial families and 30 different isolates, while Assam tea leaves obtained from Phrae province had the lowest bacterial diversity with 14 different isolates belonging to two families, *Bacillaceae* and *Staphylococcaceae*.

Interestingly, bacteria from the *Bacillaceae* and *Staphylococcaceae* families were found in all provinces, accounting for 2.7–40.9 and 4.7–84.9% of the relative abundance, respectively. The dominant bacterial families on Assam tea leaf surfaces of Chiang Mai and Nan provinces were *Microbacteriaceae* (72.8%) and *Bacillaceae* (40.9%), respectively, while the family *Staphylococcaceae* was dominant in Chiang Rai, Lampang, Phayao, and Phrae provinces with a relative abundance ranging between 37.2 and 84.9%.

When focusing on bacterial communities from leaves collected in the Nan province in March 2016 (code ML051-ML063) and March 2018 (code ML064–ML067), the data obtained suggested that the families *Micrococcaceae*, *Planococcaceae*, and *Streptococcaceae* found in 2016 were completely replaced by the *Bacillaceae* and *Staphylococcaceae* families in 2018. Likewise, in the Chiang Mai province, the families *Microbacteriaceae*, *Paenibacillaceae*, *Planococcaceae*, and *Pseudomonadaceae* found in 2015 (codes ML011–ML041) were replaced by the *Bacillaceae* and *Staphylococcaceae* families in 2016 (code ML071).

### 3.3. Bacterial Alpha Diversity, Richness, and Community Structure

The Shannon (*H’*) diversity index ranged from 0.89 to 1.49. The *H’* value of Phayao province presented the highest diversity. The Simpson’s (*D*) indices ranged between 0.51 and 0.71. Three provinces, including Chiang Mai, Chiang Rai, and Phrae provinces, had a similar *D* value of 0.53, and, consistent with *H’*, Phayao province presented the highest diversity (0.71). Likewise, Phayao province showed the highest index value of evenness (*J’* = 0.77), while the lowest value was found in Nan and Chiang Rai provinces (*J’* = 0.56). The species richness (*R*) in all provinces ranged from 18.73 to 69.84. Lampang province had the highest *R* value (69.84), whereas Phrae province showed the lowest value (18.73) (Table 1).

**Figure 4:**
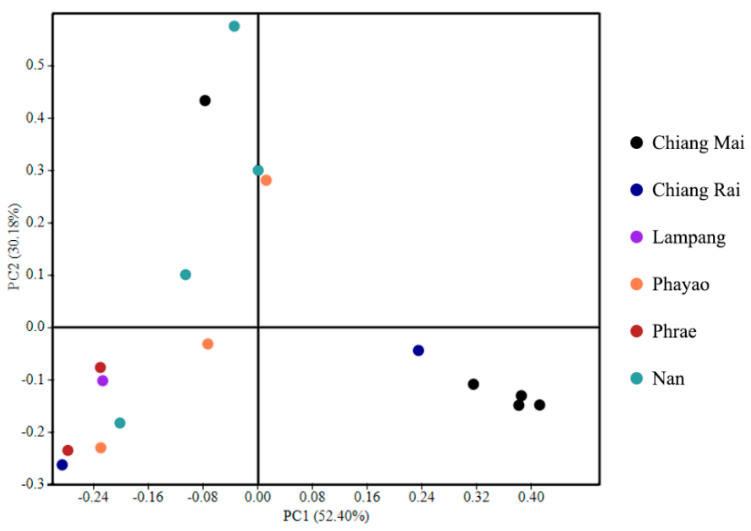
Principal coordinate analysis (PCoA) of bacterial communities on Assam tea leaves of Chiang Mai, Chiang Rai, Lampang, Phayao, Phrae, and Nan provinces.

The beta diversity analysis and the separation pattern of bacterial communities were shown by PCoA, suggesting that most bacterial communities from Chiang Mai province were explicitly separated from Lampang, Phayao, Phrae, and Nan provinces. Additionally, the bacterial communities of Chiang Rai and Lampang presented overlap between two main groups (Figure 4).

### 3.4. Probiotic Property Determination of Bacillus Strains

#### 3.4.1. Antibacterial Activity

The culture filtrates of all isolates belonging to a family *Bacillaceae* (60 isolates) were tested for their antibacterial activity against test pathogenic bacteria. *B. clausii* ML062-2 was the only isolate promoting growth inhibition of *E. coli* O157:H7 (7.2 ± 0.1 mm). *B. subtilis* ML066-3 and all *B. licheniformis* strains presented antimicrobial activity against *B. cereus* with diameters of inhibitory clear zones ranging from 7.3 ± 0.3 to 11.3 ± 0.4 mm. Three strains, namely *B. subtilis* ML066-3, *B. licheniformis* ML075-1, and *B. siamensis* ML122-2 inhibited *S. aureus* growth (8.0 to 9.0 ± 0.0 mm). All *B. siamensis* strains tested positive against MRSA (9.3–12.0 mm). Note that only *B. siamensis* ML122-2 could inhibit both *S. aureus* and MRSA (Table 2). Based on the data mentioned above, *B. licheniformis* ML075-1 and *B. siamensis* ML122-2 were selected for further probiotic potential evaluation.

#### 3.4.2. Antibiotic Susceptibility

*B. licheniformis* ML075-1 and *B. siamensis* ML122-2 were sensitive to all 16 tested antibiotics with the zone of inhibition ranging from 15.6 to 43.9 mm. Meropenem presented the widest clear zone for strain ML075-1 (41.5 mm) and ML122-2 (43.9 mm). *L. acidophilus* TISTR 2365 was susceptible to 13 antibiotics (9.9–49.0 mm of clear zone) except amikacin, co-trimoxazole, and ofloxacin (data not shown) [11].

#### 3.4.3. Survival in Gastrointestinal Tract Conditions, Autoaggregation, and Cell Surface Hydrophobicity Investigation

**Figure 5:**
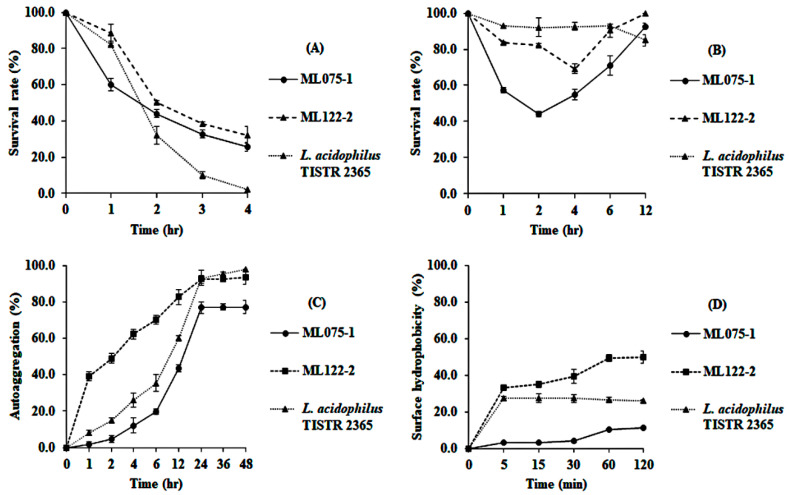
The percentage survival of *Bacillus* spp. and *L. acidophilus* TISTR 2365 in gastric (**A**) and intestinal (**B**) fluids. Autoaggregation (**C**) and cell surface hydrophobicity (**D**) abilities of strain ML075-1, ML122-2, and TISTR 2365. Data are presented as mean ± standard deviation. The experiments were done in triplicate.

*B. siamensis* ML122-2 revealed resistance to gastric fluid up to 2 h of incubation (50.4% survival rate), while *B. licheniformis* ML075-1 and *L. acidophilus* TISTR 2365 expressed 44.0 and 32.2% survival rates, respectively, for the same incubation period. *B. siamensis* ML122-2 and *B. licheniformis* ML075-1 showed resistance to the intestinal fluid for 6 h with survival rates of 90.3 and 70.9%, respectively (*p* < 0.05), while the survival rate of *L. acidophilus* TISTR 2365 was 92.7% for the same period of incubation. Fascinatingly, the survival rate of *B. licheniformis* ML075-1 and *B. siamensis* ML122-2 in intestinal fluid reached the lowest value at 2 and 4 h of incubation, respectively, and increased after further incubation for 4 and 6 h, respectively (Figure 5A,B).

For autoaggregation ability, *B. siamensis* ML122-2 presented the highest percentage of autoaggregation at 1 h after incubation (39.1%), followed by *L. acidophilus* TISTR 2365 and *B. licheniformis* ML075-1 (8.2 and 1.6%, respectively), which were significantly different (*p* < 0.05). After incubation for 24 h, *B. siamensis* ML122-2 and *L. acidophilus* TISTR 2365 had an autoaggregation percentage of 92.4 and 93.2%, respectively (*p* > 0.05), while *B. licheniformis* ML075-1 demonstrated 76.8% (*p* < 0.05). Additionally, *B. siamensis* ML122-2 showed significantly (*p* < 0.05) higher cell surface hydrophobicity than *L. acidophilus* TISTR 2365 and *B. licheniformis* ML075-1 at 30 min of incubation (39.6, 27.5 and 3.9%, respectively) (Figure 5C,D).

#### 3.4.4. Bacterial Adhesion

**Figure 6:**
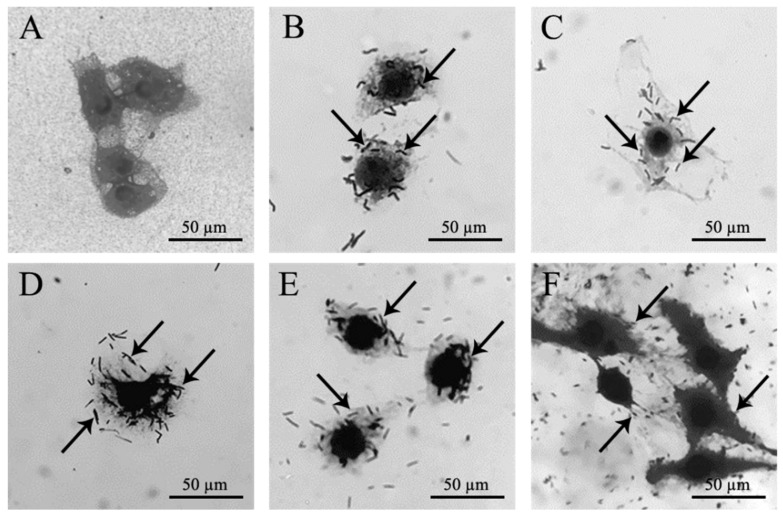
Bacterial adhesion of *B. licheniformis* ML075-1 (**B**), *B. siamensis* ML122-2 (**C**), *L. acidophilus* TISTR 2365 (**D**), *L. plantarum* FM03-1 (**E**) and *E. coli* ATCC 25922 (**F**) on Vero cells. (**A**) Vero cell control.

*B. licheniformis* ML075-1 and *B. siamensis* ML122-2 presented adhesion ability on Vero cells of 52.6 and 75.8%, respectively, when using *L. plantarum* FM03-1 as a positive control (100% adhesion). In relation to *L. acidophilus* TISTR 2365, *B. siamensis* ML122-2 (19.5%) displayed a higher adhesion on Vero cells than *B. licheniformis* ML075-1 (15.4%). Meanwhile, *E. coli* ATCC 25922 (a normal intestinal flora strain) exhibited 127.2 and 493.6% adhesion when compared with *L. acidophilus* TISTR 2365 and *L. plantarum* FM03-1, respectively (Table 3 and Figure 6).

## 4. Discussion

Assam tea plants or *Camellia sinensis* var. *assamica* are widely found in the highlands of Northern Thailand at altitudes over 200 m above sea level. The microbial composition of Assam tea leaves in Thailand first described by Rungsirivanich et al. [11] revealed the presence of culturable bacteria from the genera *Bacillus*, *Floricoccus*, *Kocuria*, *Lysinibacillus*, *Micrococcus*, and *Staphylococcus*. To gain further insight into the bacterial community of Assam tea leaves and understand their role in fermentation, as well as to identify beneficial bacteria for healthy food development and biological control, a larger area of sampling sites with various environmental conditions (altitude, soil, and climate) was explored in this study, covering six provinces (Chiang Mai, Chiang Rai, Lampang, Phayao, Phrae, and Nan) in the upper northern region of Thailand. Through molecular taxonomic markers, the isolated bacteria were classified within 15 families and 20 genera, some of which were adapted to extreme environments. For example, the genera *Anoxybacillus* and *Lysinibacillus* are reported as thermophilic [35,36], which is related to the formation of endospores. Most strains of the genus *Cellulomonas* have been described as cellulolytic bacteria, which can be found in cellulose-rich habitats [37]. On the other hand, *Pantoea* spp. *Curtobacterium* spp., *Microbacterium* spp., *Sphingomonas* spp. *Lysinibacillus* spp., *Solibacillus* spp., *Kocuria* spp., and *Pseudomonas* spp. have been grouped as endophytic bacteria implicated in plant growth promotion with some species producing antimicrobial substances [38,39,40,41,42,43,44], which may be involved in growth enhancement and biological control in Assam tea orchards [7]. The genera *Acinetobacter* and *Brevibacillus* have been reported to be found not only in the environment (involved in bioremediation [45] and biogeochemical cycles [46]) but also in animals and humans [47,48]. The *Weissella* and *Floricoccus* genera are grouped as lactic acid bacteria (LAB) [49,50]. Several reports reveal that some LAB strains can produce bacteriocin or bacteriocin-like inhibitory substances that play a role in inhibition of pathogenic microbes and in biological control. Moreover, LAB are also important in fermentation processes, and some species present probiotic properties [51]. *Bacillus* spp. have been widely found in various environments. Some species of *Bacillus* are pathogenic (e.g., *B. cereus*), probiotics (e.g., *B. clausii*, *B. subtilis*, *B. licheniformis*), endophytic (with the ability to produce the plant hormone indole-3-acetic acid or IAA; e.g., *B. altitudinis*, *B. paramycoides*, *B. tequilensis*), and antimicrobial (e.g., *B. niacin*, *B. mobilis*) [52,53,54,55,56]. *Staphylococcus* spp., *Macrococcus* spp., and *Micrococcus* spp. have been reported as normal flora on human and animal skins, and opportunistic pathogens such as *S. epidermidis*, *S. xylosus*, *Macrococcus canis,* and *Micrococcus luteus* [57,58,59] may lead to bloodstream infections. Besides that, *Corynebacterium aurimucosum* is also reported to be the cause of urinary tract infection in humans [60], while *Enterobacter hormaechei* is described as the causal agent of opportunistic infections in urinary and respiratory tracts [61]. The genus *Pantoea* has been generally found in plant surfaces as well as human feces. Some species have also been reported as human pathogens leading to bacteremia [62]. The existence of mammal microbial flora on Assam tea leaves indicates the close relationship between human, animals, and Assam tea plants. Assam tea orchards in the study area exist both in the forests and in the area around tea farmers’ houses. Possibly, the human and animal flora spread to the plants via their activities, but how these bacteria affect the tea plants is not known.

The microbial community structure has been suggested to be associated with temperature, mineralization, hydrocarbon content, soil organic matter, and moisture [63,64]. In this study, the family *Microbacteriaceae* and *Staphylococcaceae* were explicitly dominant in Chiang Mai and Phrae provinces with 72.8 and 84.9% relative abundance, respectively. Moreover, the elevation above sea level is also considered to affect microbial biodiversity [65]. Although, the number of bacterial isolates identified within the family *Bacillaceae* was higher than that of *Staphylococcaceae* (38 and 28%, respectively), the *Staphylococcaceae* family presented higher relative abundance in Chiang Rai, Lampang, Phayao, and Phrae provinces than did the family *Bacillaceae*. By interview and observation, the collecting sites in these four provinces were quite inactive in terms of tea leaf plucking for fermented Miang production during the sample collecting period. Hence, the tea plants were undisturbed, which allowed certain bacteria to establish, thrive, and succeed in this microecosystem. The active Assam tea orchards displayed higher numbers of bacterial species, possibly due to the more turns of tea leaves which were often taken off. Moreover, altitude and temperature seemed to be unrelated to the occurrence.

The microbes found on Assam tea leaf surfaces revealed an abundance of Actinobacteria, Firmicutes, and Proteobacteria phyla, which have been commonly found in the environment including soils, leaves, stems, and roots, as well as skin of human and animals [66]. In this study, all diversity indices indicated that Phayao province had the highest bacterial biodiversity, while Phrae province had the lowest. Interestingly, the family *Micrococcaceae* was not found only in Chiang Mai and Phrae provinces, which clearly displayed low diversity. The absence of the family *Micrococcaceae* in Chiang Mai and Phrae may be due to the geographical location (Figure 1). The microbial biodiversity clearly indicated the difference of bacteria existing on Assam tea leaves between two areas (referred to as the west and the east sides of Northern Thailand). The west (Mae Hong Son, Chiang Mai and Lamphun provinces) and the east (Chiang Rai, Lampang Phayao, Phrae, and Nan provinces) sides of Northern Thailand can be separated by western Phi Pan Nam range or known as Khun Tan range. Moreover, the difference of bacterial communities may be related to flow through rivers, especially the Wang River, which runs from Chiang Rai, upper Northern Thailand, passes through Lampang to Tak provinces, and lower Northern Thailand. These data support the various processes of Assam tea leaf fermentation and microbes in fermented Miang found in both areas, which have been reported by Kanpiengjai et al. [2], Khanongnuch et al. [3], and Chaikaew et al. [5].

In general, slow shifts from the original states of any microbial communities are demonstrated when continuous cultivation has been performed for a long time [7]. In this study, bacterial communities on Assam tea leaves at Nan province in March 2016 and at Chiang Mai province in 2015 were completely replaced by the *Bacillaceae* and *Staphylococcaceae* families within 2 years. This occurrence may be due to the involvement of human activity, wildfire, and deforestation in the study area and surroundings. 

“Fermented Miang” or fermented Assam tea leaves made by the local wisdom of people in the areas of Northern Thailand, the Lao People’s Democratic Republic, and the Republic of the Union of Myanmar for centuries has been unpopular with the new generation for many reasons, such as its physical appearance and old-fashioned packaging. However, the hidden market share has been noticed recently. The use of probiotics in fermented Assam tea leaves has attracted people with healthy lifestyles. Hence, information on bacterial communities would benefit further product development and elucidation of properties in the field of the gut microbiome or related subjects.

A previous study described the probiotic properties and utilization of *Bacillus* [67]. In fermented Miang, *B. tequilensis*, *B. siamensis*, *B. megaterium*, *B. toyonensis*, and *B. aryabhattai* presented great probiotic potential [68]. Some *Bacillus* strains mentioned above could be also found in this study, suggesting a persistence of these bacteria from fresh to fermented leaves.

*B. licheniformis* ML075-1 and *B. siamensis* ML122-2 demonstrated antagonistic activity against pathogenic bacteria, especially against *B. cereus* TISTR 687 and MRSA DMST 20625, respectively. Previous studies reported that *Bacillus* spp. can produce antimicrobial substances such as peptides, bacteriocins, or BLIS [16]. Interestingly, *B. siamensis* ML122-2 could inhibit growth of MRSA DMST 20625, a cause of resistance to many antibiotics. Culture broth pH seemed not to be responsible for the antagonistic activity of these isolates (data not shown). In the future, its antimicrobial active principle should be further elucidated.

In this study, *B. licheniformis* ML075-1 and *B. siamensis* ML122-2 were susceptible to all tested antibiotics. This susceptibility results may ensure that when these *Bacillus* isolates are used as probiotics, the chance that an antibiotic resistance gene will be transferred to any recipient bacteria in the intestine is scarce [15].

Acid and base tolerance are among the most important requirements for probiotics potential due to their need to survive in the stomach and intestines [69]. *B. siamensis* ML122-2 demonstrated higher survival rate in the gastric fluid than *B. licheniformis* ML075-1 and *L. acidophilus* TISTR 2365, respectively. The previous study by Kristoffersen et al. [70] indicated that *B. cereus* ATCC 14579 spores were germinated in the distal parts of the small intestine, which contains lower amounts of bile salt. In this study, the alkaline condition in simulated intestinal fluid was tested against *Bacillus* and was consistent with the previous study in that at the start of the experiment where high concentration of bile salt was present, *Bacillus* strain count was reduced, with an increasing rate after incubation for four (*B. licheniformis* ML075-1) and six (*B. siamensis* ML122-2) hours. This might be because the bacteria adapted to the test fluid or produced some metabolites to neutralize the fluid.

The adhesion ability of probiotics has been considered to be a critical prerequisite for bacterial colonization on the host gut [69]. In this study, *B. siamensis* ML122-2 demonstrated autoaggregation (higher than 90%), high hydrophobicity, and high adherence ability to Vero cells, therefore presenting the highest probiotics potential among the identified bacteria. In the future, it will be worth further investigating the potential of these species for the treatment of antibiotic-resistant infections. Furthermore, *B. siamensis* ML122-2 may provide a functional ingredient useful in synbiotic production, which will be investigated in the near future.

## 5. Conclusions

Assam tea leaves are rich in microbial biodiversity. These microbial communities may play a major role in biological control and Assam tea leaf fermentation. The microbiodiversity on Assam tea leaves presented a stabilized community representing good sources of probiotics. Particularly, *B. siamensis* ML122-2 isolated from Assam tea leaf surfaces demonstrated high probiotic potential. It represents antibacterial activity, especially MRSA; hence, its antimicrobial substance should be elucidated in the future.

## Figures and Tables

**Table 1:** Estimated number of observed diversity and richness in each province.

Province	*H’*	*D*	*J’*	*R*
Chiang Mai	0.90	0.53	0.69	26.31
Chiang Rai	1.20	0.53	0.56	58.39
Lampang	1.39	0.67	0.63	69.84
Phayao	1.49	0.71	0.77	46.74
Phrae	0.89	0.53	0.73	18.73
Nan	1.01	0.51	0.56	32.30

Abbreviations: *H’*, Shannon’s index; *D*, Simpson’s index; *J’*, Pielou’s evenness index; *R*, species richness.

**Table 2:** Antibacterial activity of *Bacillus* culture filtrates. Gentamicin, 0.1 and 50 (for MRSA) mg/mL were used as positive controls. Data were expressed as mean ± standard deviation of three independent experiments. The difference letters were considered statistically significant at *p* < 0.05.

*Bacillus* Strain	Zone of Inhibition (mm)			
	*B. cereus* TISTR 687	*E. coli* O157:H7 DMST 12743	*S. aureus* ATCC 25923	MRSA DMST 20625
*B. clausii* ML062-2	0 ^e^	7.2 ± 0.1 ^b^	0 ^d^	0 ^d^
*B. subtilis* ML066-3	7.3 ± 0.3 ^d^	0 ^c^	9.0 ± 0.0 ^b^	0 ^d^
*B. licheniformis* ML071-1	9.3 ± 0.4 ^c^	0 ^c^	0 ^d^	0 ^d^
*B. licheniformis* ML073-1	9.8 ± 0.4 ^c^	0 ^c^	0 ^d^	0 ^d^
*B. licheniformis* ML075-1	11.3 ± 0.4 ^b^	0 ^c^	9.0 ± 0.0 ^b^	0 ^d^
*B. licheniformis* ML076-2	11.0 ± 0.0 ^b^	0 ^c^	0 ^d^	0 ^d^
*B. siamensis* ML122-2	0 ^e^	0 ^c^	8.0 ± 0.0 ^c^	12.0 ± 0.0 ^a^
*B. siamensis* ML123-1	0 ^e^	0 ^c^	0 ^d^	9.3 ± 0.4 ^c^
*B. siamensis* ML124-1	0 ^e^	0 ^c^	0 ^d^	11.3 ± 0.4 ^b^
Gentamicin	16.0 ± 0.8 ^a^	11.6 ± 0.4 ^a^	14.9 ± 0.2 ^a^	12.1 ± 0.2 ^a^

**Table 3:** Bacterial adherence of *Bacillus* strains and *E. coli* ATCC 25922 to Vero cells when compared with *Lactobacillus acidophilus* and *Lactiplantibacillus plantarum*. Data are presented as mean ± standard deviation (n = 4). The different letters represent statistically significant difference (*p* < 0.05).

Bacterial Strain	Adhesion Ability (%)	
	*L. acidophilus* TISTR 2365	*L. plantarum* FM03-1
*L. acidophilus* TISTR 2365	100 ^b^	Not applied
*L. plantarum* FM03-1	Not applied	100 ^b^
*B. licheniformis* ML075-1	15.4 ± 3.3 ^c^	52.6 ± 1.6 ^d^
*B. siamensis* ML122-2	19.5 ± 1.9 ^c^	75.8 ± 7.4 ^c^
*E. coli* ATCC 25922	127.2 ± 8.2 ^a^	493.6 ± 31.8 ^a^

## Supplementary Materials

The following are available online at https://www.mdpi.com/2076-2607/8/10/1585/s1: Figure S1, Phylogenetic relationships of some bacterial isolates (bold) isolated from Assam tea leaves in Northern Thailand with their closest species and related taxa based on 16S rRNA gene sequence analysis. The branching pattern was generated by the neighbor-joining method. Bootstrap values (expressed as percentages of 1000 replications). Bar, 0.05 substitutions per nucleotide position. *Saccharolobus caldissimus* JCM 32116^T^ (GenBank accession no. LC275065) is presented as outgroup sequence. Table S1, Assam tea leaf collecting site from different regions in Northern Thailand. The data presented number of Assam tea plants, locations, altitude, bacterial cell count, number of isolates per sample, and number of species per sample. Table S2, Classification of bacteria isolated from Assam tea leaf surfaces compared with the type strain and the similarity (%) of 16S rRNA gene sequence.
