# Supplementary material for: Culturable Bacterial Community on Leaves of Assam Tea (Camellia sinensis var. assamica) in Thailand and Human Probiotic Potential of Isolated Bacillus spp
Source: Microorganisms. 2020 Oct 14;8(10):1585. doi: 10.3390/microorganisms8101585 (PMC7602384; doi:10.3390/microorganisms8101585)
Supplement: Supplementary file 1 [file microorganisms-08-01585-s001.pdf]

Supplementary Material

Culturable Bacterial Community on Leaves of Assam Tea (*Camellia sinensis* var. *assamica*) in Thailand and Human Probiotic Potential of Isolated *Bacillus* spp.

Patthanasak Rungsirivanich, Witsanu Supandee, Wirapong Futui,
Vipanee Chumsai-Na-Ayudhya, Chaowarin Yodsombat
and Narumol Thongwai

Legends of Supplementary Figure and Tables

Figure S1. Phylogenetic relationships of some bacterial isolates (bold) isolated from Assam tea leaves in Northern Thailand with their closest species and related taxa based on 16S rRNA gene sequence analysis. The branching pattern was generated by the neighbour-joining method. Bootstrap values (expressed as percentages of 1,000 replications). Bar, 0.05 substitutions per nucleotide position. *Saccharolobus caldissimus* JCM 32116^{T} (GenBank accession no. LC275065) is presented as outgroup sequence.

Table S1. Assam tea leaf collecting site from different regions in Northern Thailand. The data presented number of Assam tea plants, locations, altitude, bacterial cell count, number of isolate per sample and number of species per sample.

Table S2. Classification of bacteria isolated from Assam tea leaf surfaces compared with the type strain and the similarity (%) of 16S rRNA gene sequence.

Figure S1

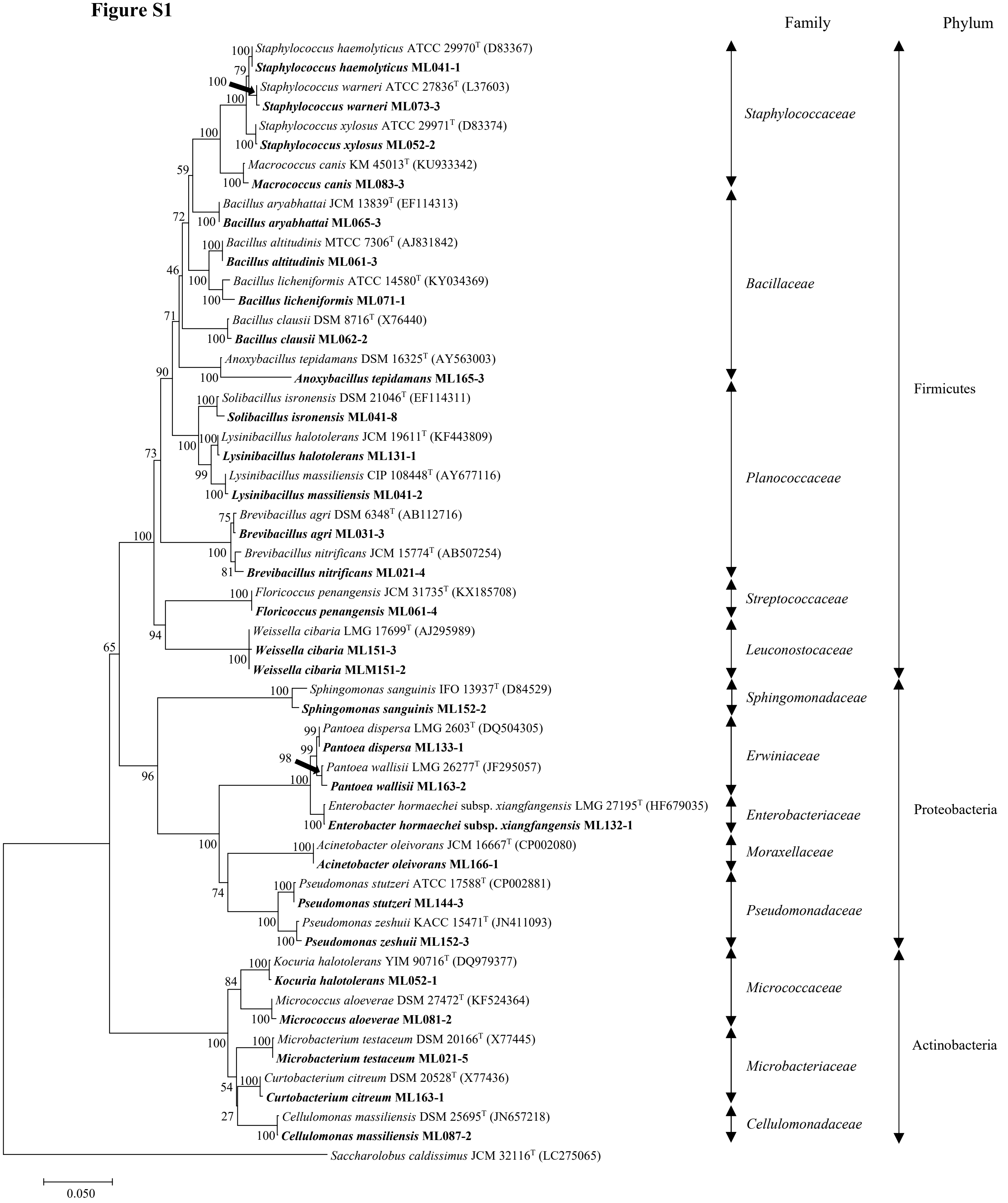
Family
Phylum
Staphylococcus haemolyticus ATCC 29970^T (D83367)
Staphylococcus haemolyticus ML041-1
Staphylococcus warneri ATCC 27836^T (L37603)
Staphylococcus warneri ML073-3
Staphylococcus xylosus ATCC 29971^T (D83374)
Staphylococcus xylosus ML052-2
Macrococcus canis KM 45013^T (KU933342)
Macrococcus canis ML083-3
Bacillus aryabhattai JCM 13839^T (EF114313)
Bacillus aryabhattai ML065-3
Bacillus altitudinis MTCC 7306^T (AJ831842)
Bacillus altitudinis ML061-3
Bacillus licheniformis ATCC 14580^T (KY034369)
Bacillus licheniformis ML071-1
Bacillus clausii DSM 8716^T (X76440)
Bacillus clausii ML062-2
Anoxybacillus tepidamans DSM 16325^T (AY563003)
Anoxybacillus tepidamans ML165-3
Solibacillus isronensis DSM 21046^T (EF114311)
Solibacillus isronensis ML041-8
Lysinibacillus halotolerans JCM 19611^T (KF443809)
Lysinibacillus halotolerans ML131-1
Lysinibacillus massiliensis CIP 108448^T (AY677116)
Lysinibacillus massiliensis ML041-2
Brevibacillus agri DSM 6348^T (AB112716)
Brevibacillus agri ML031-3
Brevibacillus nitrificans JCM 15774^T (AB507254)
Brevibacillus nitrificans ML021-4
Floricoccus penangensis JCM 31735^T (KX185708)
Floricoccus penangensis ML061-4
Weissella cibaria LMG 17699^T (AJ295989)
Weissella cibaria ML151-3
Weissella cibaria MLM151-2
Sphingomonas sanguinis IFO 13937^T (D84529)
Sphingomonas sanguinis ML152-2
Pantoea dispersa LMG 2603^T (DQ504305)
Pantoea dispersa ML133-1
Pantoea wallisii LMG 26277^T (JF295057)
Pantoea wallisii ML163-2
Enterobacter hormaechei subsp. xiangfangensis LMG 27195^T (HF679035)
Enterobacter hormaechei subsp. xiangfangensis ML132-1
Acinetobacter oleivorans JCM 16667^T (CP002080)
Acinetobacter oleivorans ML166-1
Pseudomonas stutzeri ATCC 17588^T (CP002881)
Pseudomonas stutzeri ML144-3
Pseudomonas zeshuii KACC 15471^T (JN411093)
Pseudomonas zeshuii ML152-3
Kocuria halotolerans YIM 90716^T (DQ979377)
Kocuria halotolerans ML052-1
Micrococcus aloeverae DSM 27472^T (KF524364)
Micrococcus aloeverae ML081-2
Microbacterium testaceum DSM 20166^T (X77445)
Microbacterium testaceum ML021-5
Curtobacterium citreum DSM 20528^T (X77436)
Curtobacterium citreum ML163-1
Cellulomonas massiliensis DSM 25695^T (JN657218)
Cellulomonas massiliensis ML087-2
Saccharolobus caldissimus JCM 32116^T (LC275065)
Staphylococcaceae
Bacillaceae
Planococcaceae
Streptococcaceae
Leuconostocaceae
Sphingomonadaceae
Erwiniaceae
Enterobacteriaceae
Moraxellaceae
Pseudomonadaceae
Micrococcaceae
Microbacteriaceae
Cellulomonadaceae
Firmicutes
Proteobacteria
Actinobacteria
0.050

Table S1.

Date obtained	No. of plant	Code	Location	Altitude (meter)	Collection area			Bacterial cell count (CFU/cm^2)	No. of isolate per leaf	No. of species per leaf
					Subdistrict	District	Province			
7 Jul 15	1	ML011	18°56'35.98"N, 99°21'30.07"E	1,182	Thep Sadej	Doi Saket	Chiang Mai	$1.1×10^3$	4	2
	2	ML021	18°56'14.77"N, 99°21'7.00"E	1,113	Thep Sadej	Doi Saket	Chiang Mai	$3.6×10^3$	6	5
	3	ML031	18°55'31.87"N, 99°20'22.05"E	957	Thep Sadej	Doi Saket	Chiang Mai	$3.8×10^3$	6	4
	4	ML041	18°55'17.81"N, 99°19'53.91"E	926	Thep Sadej	Doi Saket	Chiang Mai	$1.5×10^3$	7	5
12 Mar 16	5	ML051	19°12'0.76"N, 101°4'50.36"E	1,277	Pua	Pua	Nan	$5.6×10^2$	2	2
	6	ML052	19°12'0.96"N, 101°4'50.13"E	1,278	Pua	Pua	Nan	$1.3×10^3$	3	2
	7	ML061	19°15'53.62"N, 101°0'30.22"E	1,038	Sakat	Pua	Nan	$5.6×10^3$	4	4
	8	ML062	19°15'51.08"N, 101°0'31.03"E	1,030	Sakat	Pua	Nan	$2.6×10^2$	1	1
	9	ML063	19°15'49.09"N, 101°0'34.47"E	1,035	Sakat	Pua	Nan	$2.5×10^1$	2	2
24 Mar 18	10	ML064	19°16'3.63"N, 101°0'50.67"E	1,048	Sakat	Pua	Nan	$2.8×10^2$	2	2
	11	ML065	19°16'3.24"N, 101°0'51.85"E	1,055	Sakat	Pua	Nan	$1.8×10^3$	3	3
	12	ML066	19°16'7.22"N, 101°0'56.23"E	1,068	Sakat	Pua	Nan	$3.3×10^2$	3	3
	13	ML067	19°16'7.17"N, 101°0'58.05"E	1,085	Sakat	Pua	Nan	$3.8×10^2$	2	2
20 Nov 16	14	ML071	18°52'4.76"N, 99°21'14.15"E	1,130	Huay Kaew	Mae On	Chiang Mai	$6.0×10^2$	3	3
	15	ML072	18°52'4.89"N, 99°21'14.01"E	1,132	Huay Kaew	Mae On	Chiang Mai	$7.5×10^2$	3	2
	16	ML073	18°52'3.93"N, 99°21'12.02"E	1,112	Huay Kaew	Mae On	Chiang Mai	$4.7×10^3$	3	2
	17	ML074	18°51'56.83"N, 99°21'20.08"E	1,081	Huay Kaew	Mae On	Chiang Mai	$6.5×10^2$	2	1
	18	ML075	18°51'58.20"N, 99°21'19.06"E	1,057	Huay Kaew	Mae On	Chiang Mai	$2.1×10^3$	2	2
	19	ML076	18°51'57.92"N, 99°21'17.06"E	1,057	Huay Kaew	Mae On	Chiang Mai	$1.9×10^4$	3	3
11 Feb 18	20	ML081	18°50'4.05"N, 99°23'18.52"E	1,066	Chae Son	Mueang Pan	Lampang	$1.5×10^3$	2	2
	21	ML082	18°50'3.64"N, 99°23'17.51"E	1,040	Chae Son	Mueang Pan	Lampang	$9.5×10^3$	5	5
	22	ML083	18°50'5.00"N, 99°23'21.74"E	1,065	Chae Son	Mueang Pan	Lampang	$6.0×10^3$	3	3
	23	ML084	18°50'1.64"N, 99°23'16.81"E	1,045	Chae Son	Mueang Pan	Lampang	$2.5×10^1$	1	1
	24	ML085	18°50'2.41"N, 99°23'16.26"E	1,045	Chae Son	Mueang Pan	Lampang	$2.5×10^1$	1	1
	25	ML087	18°49'50.39"N, 99°23'11.31"E	1,008	Chae Son	Mueang Pan	Lampang	$1.5×10^3$	2	2
23 Mar 18	26	ML091	18°43'45.37"N, 100°49'57.38"E	243	Nam Kian	Phu Phiang	Nan	$3.5×10^2$	1	1
	27	ML093	18°43'45.25"N, 100°49'57.11"E	243	Nam Kian	Phu Phiang	Nan	$2.5×10^1$	1	1
	28	ML094	18°43'46.41"N, 100°49'55.32"E	243	Nam Kian	Phu Phiang	Nan	$7.5×10^1$	2	1
	29	ML095	18°43'46.24"N, 100°49'55.24"E	243	Nam Kian	Phu Phiang	Nan	$1.0×10^2$	1	1
	30	ML096	18°43'46.48"N, 100°49'54.21"E	243	Nam Kian	Phu Phiang	Nan	$1.2×10^3$	1	1
24 Mar 18	31	ML101	18°47'34.13"N, 100°38'0.74"E	376	Rueang	Mueang Nan	Nan	$5.8×10^2$	3	2
	32	ML102	18°47'34.79"N, 100°38'0.82"E	376	Rueang	Mueang Nan	Nan	$1.3×10^2$	1	1
	33	ML103	18°47'32.07"N, 100°38'0.49"E	389	Rueang	Mueang Nan	Nan	$1.5×10^3$	3	3
	34	ML104	18°47'32.31"N, 100°37'59.82"E	388	Rueang	Mueang Nan	Nan	$1.8×10^2$	2	2
	35	ML106	18°47'33.05"N, 100°37'58.80"E	384	Rueang	Mueang Nan	Nan	$4.0×10^2$	2	1
25 Mar 18	36	ML111	18°0'1.19"N, 100°15'22.01"E	623	Cho Hae	Mueang Phrae	Phrae	$2.5×10^1$	1	1
	37	ML112	18°0'0.81"N, 100°15'21.88"E	621	Cho Hae	Mueang Phrae	Phrae	$4.5×10^2$	2	2
	38	ML113	18°0'7.00"N, 100°15'20.57"E	648	Cho Hae	Mueang Phrae	Phrae	$6.8×10^2$	2	2

Table S1. (continued)

Date obtained	No. of plant	Code	Location	Altitude (meter)	Collection area			Bacterial cell count (CFU/cm^2)	No. of isolate per leaf	No. of species per leaf
					Subdistrict	District	Province			
25 Mar 18	39	ML114	18°0'8.46"N, 100°15'18.82"E	652	Cho Hae	Mueang Phrae	Phrae	1.4×10^3	3	3
	40	ML121	18°0'46.74"N, 100°16'50.91"E	599	Cho Hae	Mueang Phrae	Phrae	2.7×10^3	2	2
	41	ML122	18°0'45.21"N, 100°16'51.79"E	608	Cho Hae	Mueang Phrae	Phrae	3.1×10^3	2	2
	42	ML123	18°0'44.54"N, 100°16'53.49"E	607	Cho Hae	Mueang Phrae	Phrae	1.0×10^2	1	1
	43	ML124	18°0'44.89"N, 100°16'53.73"E	604	Cho Hae	Mueang Phrae	Phrae	7.5×10^1	1	1
16 Nov 18	44	ML131	18°55'47.05"N, 100°5'25.75"E	629	Nonglom	Dok Khamtai	Phayao	6.5×10^2	3	3
	45	ML132	18°55'47.18"N, 100°5'25.68"E	628	Nonglom	Dok Khamtai	Phayao	8.3×10^2	1	1
	46	ML133	18°55'47.79"N, 100°5'25.00"E	627	Nonglom	Dok Khamtai	Phayao	5.4×10^3	3	3
17 Nov 18	47	ML142	19°23'46.83"N, 99°41'55.05"E	950	Charoenrat	Mae Chai	Phayao	1.3×10^3	2	2
	48	ML143	19°23'47.00"N, 99°41'55.22"E	951	Charoenrat	Mae Chai	Phayao	7.8×10^2	2	2
	49	ML144	19°23'47.36"N, 99°41'55.11"E	952	Charoenrat	Mae Chai	Phayao	5.3×10^2	4	4
	50	ML145	19°23'47.68"N, 99°41'55.27"E	950	Charoenrat	Mae Chai	Phayao	2.2×10^3	3	3
	51	ML146	19°23'47.34"N, 99°41'54.91"E	954	Charoenrat	Mae Chai	Phayao	2.5×10^3	4	3
	52	ML151	19°21'13.31"N, 99°42'48.01"E	828	Sritoi	Mae Chai	Phayao	8.3×10^2	4	3
	53	ML152	19°21'13.20"N, 99°42'48.45"E	825	Sritoi	Mae Chai	Phayao	3.8×10^2	3	3
	54	ML153	19°21'13.26"N, 99°42'48.02"E	828	Sritoi	Mae Chai	Phayao	5.0×10^3	2	2
	55	ML163	19°34'51.58"N, 100°2'42.41"E	399	Thoeng	Mae Loi	Chiang Rai	5.6×10^3	4	4
	56	ML165	19°34'51.68"N, 100°2'42.22"E	399	Thoeng	Mae Loi	Chiang Rai	4.5×10^3	4	4
	57	ML166	19°34'54.77"N, 100°1'50.34"E	436	Thoeng	Mae Loi	Chiang Rai	3.9×10^3	2	2
	58	ML167	19°34'54.17"N, 100°1'50.32"E	438	Thoeng	Mae Loi	Chiang Rai	1.5×10^3	2	2
18 Nov 18	59	ML171	19°5'30.66"N, 99°22'17.86"E	1,080	Mae Chedi	Wiang Papao	Chiang Rai	2.8×10^2	2	2
	60	ML172	19°5'30.30"N, 99°22'18.18"E	1,077	Mae Chedi	Wiang Papao	Chiang Rai	2.0×10^2	2	2
	61	ML173	19°5'30.55"N, 99°22'19.09"E	1,080	Mae Chedi	Wiang Papao	Chiang Rai	8.3×10^2	2	2
	62	ML174	19°5'30.78"N, 99°22'18.19"E	1,081	Mae Chedi	Wiang Papao	Chiang Rai	5.3×10^2	2	2

Figure S2.

Family	Species	Number of isolate found	Type strain	Similarity (%)
Bacillaceae	*Anoxybacillus tepidamans*	2	DSM 16325	92.4-95.3
	Bacillus altitudinis	1	MTCC 7306	99.9
	Bacillus aryabhattai	4	JCM 13839	100.0
	Bacillus cereus	3	ATCC 14579	99.9
	Bacillus clausii	1	DSM 8716	99.7
	Bacillus licheniformis	5	ATCC 14580	98.4-99.4
	Bacillus megaterium	1	ATCC 14581	99.9
	Bacillus mobilis	1	MCCC 1A05942	100.0
	Bacillus niacin	1	IFO 15566	99.0
	Bacillus paramycoides	1	LMG 28876	99.9
	Bacillus siamensis	24	KCTC 13613	99.7-99.9
	Bacillus subtilis subsp. *inaquosorum*	2	DSM 22148	99.6-99.9
	Bacillus subtilis subsp. *subtilis*	4	JCM 1465	99.7-99.9
	Bacillus tequilensis	10	NCTC 13306	98.3-99.9
Staphylococcaceae	*Macrococcus canis*	1	KM 45013	99.4
	Macrococcus goetzii	3	CCM 4927	99.3-99.4
	Staphylococcus cohnii subsp. *cohnii*	1	ATCC 29974	99.9
	Staphylococcus epidermidis	7	ATCC 14990	99.5-100.0
	Staphylococcus haemolyticus	18	ATCC 29970	99.5-100.0
	Staphylococcus hominis subsp. *hominis*	10	DSM 20328	99.5-99.9
	Staphylococcus hominis subsp. *novobiosepticus*	2	GTC 1228	99.9
	Staphylococcus warneri	1	ATCC 27836	99.8
	Staphylococcus xylosus	1	ATCC 29971	99.9
Microbacteriaceae	*Curtobacterium citreum*	2	DSM 20528	99.6-100.0
	Curtobacterium luteum	4	DSM 20542	99.7-99.8
	Curtobacterium oceanosedimentum	11	ATCC 31317	97.7-99.9
	Microbacterium testaceum	1	DSM 20166	99.8
Micrococcaceae	*Kocuria halotolerans*	1	YIM 90716	99.7
	Micrococcus aloeverae	8	DSM 27472	90.2-99.8
	Micrococcus luteus	1	DSM 20030	93.2
	Micrococcus yunnanensis	1	YIM 65004	98.8
Planococcaceae	*Lysinibacillus contaminans*	1	DSM 25560	97.9
	Lysinibacillus halotolerans	1	JCM 19611	99.9
	Lysinibacillus massiliensis	1	CIP 108448	99.8
	Solibacillus isronensis	1	DSM 21046	99.4
Erwiniaceae	*Pantoea brenneri*	1	LMG 5343	99.6

Figure S2. (continued)

Family	Species	Number of isolate found	Type strain	Similarity (%)
Erwiniaceae	*Pantoea dispersa*	1	LMG 2603	100.0
	Pantoea wallisii	1	LMG 26277	99.3
Paenibacillaceae	*Brevibacillus agri*	1	DSM 6348	87.9
	Brevibacillus nitrificans	1	JCM 15774	98.6
Pseudomonadaceae	*Pseudomonas stutzeri*	5	ATCC 17588	98.4-99.9
	Pseudomonas zeshuii	1	KACC 15471	99.6
Cellulomonadaceae	*Cellulomonas massiliensis*	1	DSM 25695	99.7
Corynebacteriaceae	*Corynebacterium aurimucosum*	2	DSM 44532	96.7-98.1
Enterobacteriaceae	*Enterobacter hormaechei* subsp. *xiangfangensis*	1	LMG 27195	99.7
Leuconostocaceae	*Weissella cibaria*	2	LMG 17699	99.8-99.9
Moraxellaceae	*Acinetobacter oleivorans*	1	JCM 16667	100.0
Sphingomonadaceae	*Sphingomonas sanguinis*	1	IFO 13937	99.6
Streptococcaceae	*Floricoccus penangensis*	1	JCM 31735	100.0
